# Comparative assessment of the efficacy of an intralesional injection of placentrex, hyaluronidase and dexamethasone in the management of oral submucous fibrosis: A randomized controlled trial

**DOI:** 10.3892/mi.2024.143

**Published:** 2024-02-26

**Authors:** Jay Goyal, Shruthi Iyer, Chinmayee Palande, Ujwala Brahmankar, Janice John, Kshitija Patil

**Affiliations:** 1 Department of Oral Surgery, Jawahar Medical Foundation's ACPM Dental College, Dhule, Maharashtra 424001, India; 2 Department of Oral Medicine, Jawahar Medical Foundation's ACPM Dental College, Dhule, Maharashtra 424001, India

**Keywords:** placentrex, hyaluronidase, dexamethasone, oral submucous fibrosis, trismus, burning sensation

## Abstract

The prevalent symptoms of oral submucous fibrosis (OSMF) are a burning sensation and trismus. The aim of the present study was to compare the efficacy of placentrex, hyaluronidase and dexamethasone, and their combination in the treatment of OSMF. For this purpose, 160 patients with OSMF were divided into four groups (each with 40 patients at a 1:1:1:1 allocation ratio). The patients in group 1 (control) received only oral supplements, along with regular mouth-opening exercises; patients in group 2 received an injection of placental extract; patients in group 3 were injected with hyaluronidase and dexamethasone; and patients in group 4 received a combination of injections from groups 2 and 3. The injections were administered once weekly for 12 weeks and patients were followed-up for 12 months. The data of the patients (mouth opening ability and a burning sensation) were analyzed using ANOVA and the Kruskal-Wallis test. The maximum increase in mouth opening (7.30±0.80 mm) was noted in group 4, and the lease increase was observed in the control group (0.37±0.16 mm), from baseline levels to the end of the 12th week. The maximum relapse in mouth opening of 1.62±0.45 mm was noted in group 2, and a minimum relapse of 0.20±0.08 mm was noted in group 4. On the whole, the present study demonstrates that the intralesional injection of a combination of the three drugs (placentrex, hyaluronidase and dexamethasone) in addition to the use of oral supplements and mouth opening exercises has a high level of efficacy in improving trismus and burning sensation in patients with OSMF.

## Introduction

Oral submucous fibrosis (OSMF) is a chronic disease of uncertain origin that has a gradual and harmful effect on the mucous membranes of various regions within the oral cavity. Furthermore, it can sporadically spread to the pharynx and esophagus and, on rare occasions, even to the larynx. It is characterized by a sub-epithelial inflammatory reaction followed by progressive fibrosis, leading to trismus and epithelial atrophy (1). It is commonly observed in individuals who are 20-35 years of age, and mostly in India and Southeast Asia (prevalence rate, 2.26%), where it is commonly associated with the use of areca nuts (2).

The etiology of the disease is exceedingly intricate, and none of the therapeutic approaches employed to date have yielded adequate outcomes. The management of patients with OSMF is dependent on the extent of disease progression and its clinical manifestations. In the initial phases, the cessation of deleterious habits and the administration of nutritional supplements (multivitamins) are implemented. During the intermediate stages, a conservative approach is adopted which entails intralesional injections in conjunction with medical therapy. Surgical intervention is imperative in the advanced stages of the disease (3). The chief concern of affected patient is invariably the reduction in mouth opening (trismus) and associated discomfort with the consumption of food due to the burning sensation. However, the main concern of clinicians is the potential of the disease for malignant transformation (1).

The main goal of treatments for OSMF is to reduce the associated burning sensation and trismus. This can be achieved by therapy with intralesional injections of placental extract, hyaluronidase and corticosteroids. These treatments have been used alone or in combination in previous studies (4-8). However, it should be noted that, to the best of our knowledge, none of the studies published to date have considered the inclusion of a control group. Moreover, it is worth mentioning that only two studies have undertaken a brief follow-up examination spanning a period of 9 months (5,6). Furthermore, it is important to highlight that only two studies have been conducted with a sample size of 60 patients, but utilize only two medications and no follow-up was performed (7,8). Therefore, the present study was undertaken in an aim to compare the therapeutic outcomes of patients with OSMF treated with an intralesional injection of placental extract, hyaluronidase combined with dexamethasone, and placental extract combined with hyaluronidase and dexamethasone, in comparison to a control group. In addition, the present study analyzed the loss of mouth opening 1 year following the cessation of treatment. The null hypothesis posited in the present study suggests that there is no disparity in the therapeutic effects of the three modalities in patients with OSMF.

## Patients and methods

### Study overview

**Figure 1:**
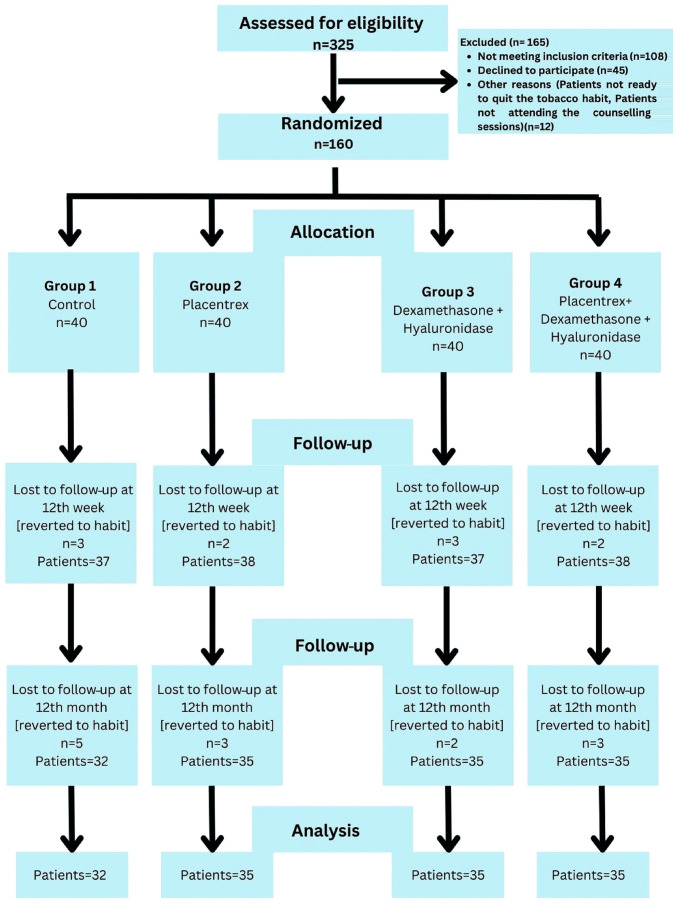
Flowchart of the selection and exclusion protocol in the present study.

A single-center, parallel-group, randomized controlled trial (RCT) with a 1:1:1:1 allocation ratio (40 patients in each group) was conducted at the Department of Oral Surgery, Jawahar Medical Foundation's ACPM Dental College, Dhule, India between March, 2022 and September, 2023. The patients presented with trismus, a blanched, white marble-like appearance of the oral mucosa, palpable fibrous bands, sunken cheeks, difficulty in eating and a mild to moderate burning sensation. A detailed history of the use of areca nuts, tobacco and its related products, as well as the consumption of alcohol, and hot and spicy food was obtained from the participants. Patients who were willing to participate were examined thoroughly by a senior oral surgeon for the presence of clinical symptoms, and the confirmation of OSMF was performed by a histopathological examination. The study was conducted according to the principles of the Declaration of Helsinki. The present RCT was conducted according to the Consolidated Standards of Reporting Trials (CONSORT) guidelines, which are designed to improve the reporting of parallel-group randomized controlled trials (9) as shown in Fig. 1. This trial was registered at the Clinical Trial Registry of India at www.ctri.nic.in (CTRI/2022/01/061403; https://ctri.nic.in/Clinicaltrials/pmaindet2.php?EncHid=OTY1NDk=&Enc=&userName=).

### Ethical considerations

The present study was approved by the Institutional Ethical Committee of Jawahar Medical Foundation Annasaheb Chudaman Patil Memorial Dental College (EC/NEW/INST/2022/2959/2022/63). Written informed consent was obtained from all the patients after explaining the outcomes and potential complications of the procedure. The control group signed the consent forms stating that they did not wish to receive treatment for intra-lesional injections and therefore, patients in the control group were provided with oral supplements for OSMF, such as antioxidants (OSMF Vita, Sterlife Biotec), 0.1% triamcinolone oromucosal paste (Turbocort, Indoco Remedies Ltd.), and 0.15% benzydamine mouthwash (B-mine, Dupen Laboratories Pvt. Ltd.), along with regular mouth opening exercises.

### Sample size calculation

The sample size was calculated using G*Power statistical software (Ver. 3.1 Franz Faul, Universität Kiel, Kiel, Germany). The sample size was calculated from a previous study with a type I error of 0.05, power of 80%, and an effect size of 0.3; the resulting sample size was 15 for each group (4). The present study was conducted with 40 patients in each group, considering a 10% dropout rate and increasing the power of the study to 90%.

### Study population

A total of 325 patients with OSMF were screened based on the eligibility criteria, and 160 patients were selected for inclusion in the study based on the inclusion and exclusion criteria. Patients diagnosed with grade 2 and grade 3 OSMF, as evidenced from their results of the detailed clinical and histopathological examination, according to the classification protocol described in the study by Passi *et al* (10), were included in the study.

Patients previously treated for OSMF; patients who had grade 1 and 4 OSMF; those who were allergic to placental extracts, steroids and hyaluronidase; those who did not give their consent; those with syndromic or systemic disease with mouth ulcers; those who had undergone radiotherapy sessions leading to oral ulcers; and those who had a previous history of major surgery in the oral cavity or carcinoma of the oral cavity were excluded from the study.

Routine hematological examinations, such as complete blood count, clotting analysis, serum electrolyte assessment, erythrocyte sedimentation rate (ESR), vitamin B12 levels, human immunodeficiency virus detection and hepatitis B surface antigen evaluation were conducted during the initial visit of the patients.

The 160 patients with grade 2 and 3 OSMF who were included in the study (Fig. 1) were divided into four groups as follows: Group 1, 40 patients who did not agree to receive intralesional injections, and were therefore administered oral medication for OSMF; group 2, 40 patients who received an intralesional injection of 2 ml placental extracts (Placentrex, Albert David Pharmaceuticals Ltd.), mixed with 1 ml of 2% lignocaine; group 3, 40 patients who received an injection of 1,500 IU of 1 ml hyaluronidase (Hynidase, Shreya Pharmaceuticals Ltd.), with 1 ml dexamethasone (Decadron, Wockhardt Ltd.), mixed with 1 ml of 2% lignocaine; group 4, 40 patients who received a combination of the drugs in the aforementioned two groups (2 ml placental extracts, 1 ml hyaluronidase, 1 ml dexamethasone and 1 ml of 2% lignocaine).

Randomization was exclusively performed for the experimental groups, while the control group, due to ethical considerations, comprised individuals who declined the intra-lesional injection treatment. A computerized random number generator was used to generate a sequence lacking a particular pattern (Stat-Trek program; https://stattrek.com/). A total of 120 non-transparent envelopes (for only the experimental groups) were meticulously assembled and placed in a bowl. These envelopes were methodically assigned a code and securely sealed by a separate researcher who was not involved in the patient selection. Each participant in the experimental group was instructed to select an envelope. Subsequently, each participant was allocated an intervention in accordance with the selected envelope.

The present study was an open-label study in which the patients in the control group and the oral surgeon who treated the patients could not be blinded. In the context of an open-label investigation, it is desirable for the evaluation of the results to be concealed, if feasible. Consequently, the statistician was unaware of the allocation of the individuals to their respective groups. Given the ethical considerations, it was not possible to conceal the control group. To minimize any potential bias in the evaluation process, the patients participating in the experimental groups were not made aware of the specific pharmacological agent employed for the intra-lesional injections.

### Procedure

Patients were actively discouraged (through preliminary counseling session) from consuming areca nut and tobacco products, alcohol, and hot and spicy foods, which are strongly associated with the development of the condition under consideration. Furthermore, the patients were advised to adopt a well-balanced diet. Moreover, meticulous efforts were made to eliminate any potential sources of infection and irritation within the oral cavity, such as the enameloplasty of sharp cusps, thus ensuring a comprehensive approach for the management of the condition. The patients were provided with oral prophylaxis, which involved thorough cleaning and the maintenance of oral hygiene to mitigate the risk of further complications. Repeated counseling sessions were conducted with the patients to motivate them to leave the habit of using tobacco products, alcohol, and hot and spicy foods. Following a thorough assessment after a period of 8 weeks, the patients were required to provide assurance that they had successfully refrained from engaging in deleterious habits as instructed, and based on their compliance, further management and treatment were continued.

Drugs were introduced into the submucosal layer at the affected site, mainly in the retromolar area (area of maximum fibrosis), using an insulin syringe and needle, with the aim of mitigating the development of fibrotic tissue that can result from repeated injections. The patients were instructed to abstain from rinsing their oral cavity for a minimum of 1 h subsequent to receiving the aforementioned trio of submucosal injections. The inclusion of lignocaine was intended to alleviate irritation and enhance the drug distribution. The injections were administered bi-weekly for a period of 3 months. All patients were also administered SM fibro tablets (Warren Pharmaceuticals, Pvt. Ltd.) containing zinc, selenium, copper, β-carotene, α-lipoic acid, vitamin E and lycopene, to be taken twice daily for 1 year. The patients were also instructed to perform mouth opening exercises regularly with ice cream sticks five times a day, to perform massages with 0.1% triamcinolone oromucosal paste (Turbocort, Indoco Remedies Ltd.) twice daily and to rinse with benzydamine 0.15% mouthwash (B-mine Dupen Laboratories Pvt. Ltd.) for 1 year (11). All patients were followed-up at monthly visits, and repeated reminders were sent to their mobile phones to remind them to follow the prescription diligently and refrain from using areca nuts, tobacco products, alcohol, and hot and spicy foods.

The primary outcome of the study was to assess the improvement in mouth opening with the intervention compared to the control group. Measurements of mouth opening in millimeters were conducted using a disposable measuring scale. Interincisal mouth opening was determined by measuring the distance between the mesioincisal angle of the upper central incisor and the mesioincisal angle of the lower central incisor. A standardized protocol was followed for all cases of OSMF (12). The secondary outcome was the assessment of the improvement in the burning sensation felt by the patients. A five-point Likert numeric scale was used to assess the burning sensation (0, indicated no burning sensation; 1, indicated a mild burning sensation; 2, indicated a moderate burning sensation; 3, indicated a severe burning sensation; and 4, indicated an extreme burning sensation) (13). All measurements were obtained at baseline, and at the 4th, 8th and 12th week, and 12 months following intervention, as described in previous studies (4,6,7).

### Statistical analysis

The collected data were statistically analyzed by the descriptive analysis of mean, range and standard deviation using SPSS software version 22 (IBM Corp.). The Shapiro-Wilk test was used to examine the normality of the data. For normally distributed data, parametric tests, such as one-way ANOVA were used for inter-group comparisons of the interincisal distance between the groups, and repeated ANOVA was used for intra-group comparisons, followed by post-hoc analysis using Tukey's test. For data which were not normally distributed, non-parametric tests, such as the Kruskal-Wallis test, was used for inter- and intra-group comparisons of burning sensation between the groups, followed by post-hoc analysis using Dunn's test. A value of <0.05 was considered to indicate a statistically significant difference.

## Results

The baseline characteristics of the groups are presented in Table I. All groups exhibited non-significant differences as regards the demographic characteristics of the patients, such as age, sex distribution, and patients with grade 2 and 3 OSMF, which nullified the confounding bias due to these factors (P>0.05). The number of patients with grade 2 OSMF was greater than that of those with grade 3 OSMF. All the males were mostly tobacco chewers, and few were betal quid chewers (88%, 12% respectively), whereas all the females were betal quid chewers. In total, 55% of the patients complained of the presence of a white patch in their mouth, 88% complained of reduced mouth opening, and 11% complained of a burning sensation. The routine blood investigations yielded results which were within the normal range in the majority of the patients (96%); only 5 patients had an iron deficiency (mean value, 42±10.5 µg/dl), 3 patients had an increased ESR (23.15±3.5 mm/h) and 4 patients had a vitamin B12 deficiency (mean value, 225.12±23.12 pg/ml). Although the study initially enrolled 40 patients, a subset of patients was ultimately excluded from the analysis at the conclusion of the 1-year follow-up period. This exclusion was due to their resumption of the habit of consuming areca nut or tobacco products, as well as to their discontinuation of treatment attendance, despite repeated reminders and counseling efforts.

Statistically significant differences were noted between the groups for improvement in mouth opening and burning sensation, and therefore, the null hypothesis was rejected. The mean mouth opening increased in all groups, with highly statistically significant differences observed in the experimental groups (P<0.001) and non-significant differences were noted in the control group (P=0.067), from baseline to the end of the follow-up period at 1 year. There was a gradual increase in mouth opening in all groups from baseline to the 12th week of treatment, although this relapsed by the end of 1year. A maximum increase of 7.30±0.80 mm was noted in group 4, followed by 5.94±0.84 mm in group 2, 5.11±0.92 mm in group 3, and least in the control group (group 1) of 0.37±0.16 mm, from baseline to the end of 12th week. No significant differences were observed between the groups at baseline. This indicated that all groups were comparable at baseline with similar mouth opening, which nullified the confounding bias due to variable differences in the groups at baseline (P=0.85). The maximum increase in mouth opening was noted at the 12th week in group 4, followed by group 2, and the lowest in the control group. The maximum relapse of 1.62±0.45 mm was observed in group 2, followed by 0.52±0.83 mm relapse in group 3, and a minimum relapse of 0.20±0.08 mm was observed in group 4, as shown in Table II.

Upon further comparison of the groups by post-hoc analysis at the 12th week, it was noted that non-significant differences were observed between groups 2 and 3 as regards the increase in mouth opening, whereas statistically significant differences were found between the control group and the experimental groups, as shown in Table III.

Groups 1, 2 and 3 exhibited no improvement in burning sensation at 1 month of treatment, and thereafter, exhibited a decrease until 1 year of follow-up. Group 4 exhibited a decrease in burning sensation from baseline until 1 year of follow-up. At baseline, all groups were similar, with no significant differences, which nullified the confounding bias of variable differences in the groups. The maximum reduction in burning sensation was observed in group 4, followed by groups 3 and 2, and the least reduction was observed in the control group, as shown in Table IV. Post-hoc analysis using Dunn's test at the 12th week of follow-up revealed that the patients in whom placental extract injection was administered exhibited non-significant differences in the reduction of burning sensation when compared to patients in whom a mixture of dexamethasone and hyaluronidase was administered. However, intralesional injections of medications significantly reduced the burning sensation compared with the control group, as shown in Table V.

## Discussion

OSMF is highly prevalent among the Indian population. Given its potential for premalignancy (5.6%) and the severity of clinical manifestations, numerous studies have been conducted by various authors to investigate different facets of this condition, such as scleroderma, including its etiology, pathogenesis and treatment. However, the precise etiology and pathogenesis of OSMF remain unclear (1-3). Chewing areca nuts is the most prevalent causal factor for OSMF. The predominant practice involves placing a betel quid in the vestibule for various durations of time and frequencies. The continuous interaction between betel quid and the oral mucosa leads to the absorption and metabolic processing of quid alkaloids and flavonoids. These components, along with their metabolites, act as persistent sources of irritation in the oral mucosa. Furthermore, the inclusion of coarse fibers in betel quid results in mechanical irritation of the oral mucosa. The resulting microtrauma caused by the ongoing friction of these coarse fibers from the areca nut facilitates the diffusion of betel quid alkaloids and flavonoids into the connective tissue beneath the epithelium, leading to infiltration of inflammatory cells into the juxta-epithelial region (3).

In the present study, the majority of the males were tobacco chewers (88%), and all the females were betel quid chewers; similar findings were observed in a study conducted on Indian females (3). The history of habits ranged from a minimum of 2 to 5 years in the majority of patients, which depicts the slow, progressive nature of OSMF (2). Patients chew tobacco or betel quid to 4-5 times per day. The chief complaint of the patients was a reduction in mouth opening (88%), and some patients complained of a mild to moderate burning sensation (11%).

The initial symptoms of OSMF are a burning sensation, pain and ulceration, which are predominantly observed in patients with grade 1 OSMF. In the present study, as the included patients had grade 2 and 3 OSMF, it was revealed that the majority of the patients had a mild to moderate burning sensation (63% of the patients had grade 2 OSMF, and 37% of the patients had grade 3 OSMF). This may be due to the presence of a maximum number of degranulated mast cells in the initial stages of OSMF, which decreases as the stage of OSMF progresses (14). With the advancing stage of OSMF, fibrosis increases in the underlying connective tissues, leading to reduced mouth opening. The majority of the patients had reduced mouth opening and fibrosis with blanching of the oral mucosa. This may be due to an increase in collagen synthesis as a result of the active components of betel nuts. Recently, exosomes originating from mesenchymal stem cells obtained from human adipose tissue have demonstrated the ability to enhance the proliferative and migratory capabilities of myofibroblasts, while also impeding the deposition of collagen and the process of trans-differentiation *in vitro*. Exosomes regulate the TGF-β pathway within myofibroblasts and exhibit antifibrotic properties, rendering them a potentially favorable therapeutic modality for treating OSMF (15).

In the present study, few patients with OSMF had an increased ESR, iron and vitamin B12 deficiency, as also observed in a previous study (16). OSMF can be identified as a collagen metabolic disorder that arises from the excess production of extensively interconnected insoluble collagen type I. Collagen hydroxylation requires the presence of ferrous iron and ascorbic acid. The utilization of iron in the hydroxylation of proline and lysine results in a reduction in serum iron levels, which further leads to a decrease in vascularity and facilitating fibrosis (17). Iron and Vitamin B12 deficiencies arise mainly due to impaired food intake, resulting from ulceration and a burning sensation in the initial stages of OSMF, leading to an impairment in the inflammatory reparative response of the lamina propria, which ultimately leads to the progression of OSMF (18). Therefore, patients with an iron or Vitamin B12 deficiency were administered supplements apart from other prescribed interventions.

In the present study, the local administration of placental extracts led to a significant improvement in mouth opening by 5.94±0.84 mm after 12 weeks of treatment, but relapsed by 1.62±0.45 mm after 1 year follow-up. These findings are consistent with those of previous studies (6,13). Placental extracts contain growth factors (TGF, FGF and VEGF) that can reduce inflammation and inhibit platelet aggregation. The activity of placental extract primarily involves the stimulation of biogenesis, and its application is based on the principles of tissue therapy. According to this hypothesis, tissues undergo a process of biogenic adaptation, resulting in the generation of substances necessary for their sustenance and vitality through biogenic stimulation (13).

In the present study, the injection of dexamethasone and hyaluronidase resulted in improvement of mouth opening by 5.11±0.92 mm after 12 weeks of treatment. Although it was not significantly different from the placental extract group, a relapse of 0.52±0.83 mm only was noted after 1 year of follow-up, which was less than that in the placental extract group (1.62±0.45 mm). The improvement in the burning sensation was also greater than that in the control group. These findings are consistent with those of previous studies (4,5,8). However, these findings contradict those of Kisave *et al* (7) who reported a statistically significant improvement in mouth opening by hydrocortisone, compared to placentrex. The disparity in the results may be due to the differences in methodology. Dexamethasone plays a crucial role in the suppression of the immune system by reducing the activity and volume of the lymphatic system. By inhibiting the migration of polymorphonuclear leukocytes and reversing capillary permeability, it effectively diminishes inflammatory components and burning sensations in patients with OSMF. As a corticosteroid for intralesional injections, it demonstrates superior local potency, a prolonged duration of action and minimal systemic absorption. Steroids contribute to the initial alleviation of symptoms in patients with limited mouth opening by clearing juxta-epithelial inflammation and promoting collagen formation (19). Hyaluronidase is an enzyme that diminishes the density of the ground substance and consequently augments the permeability of tissues to injected corticosteroids. It stimulates the hydrolysis of hyaluronic acid, a major component of tissue cement, which hinders the dispersion of fluids through tissues. Furthermore, it facilitates the dispersion and assimilation of injection locally (20).

The present study indicated that the combination of placental extracts, dexamethasone and hyaluronidase led to a maximum improvement of 7.30±0.80 mm in mouth opening, with a minimum relapse of 0.20±0.08 mm after 1 year of follow-up. The maximum improvement in burning sensation was also observed in this group (group 4). This may be due to the synergistic effects of various drugs used in combination. These findings are in agreement with those of the study by Shrinivas *et al* (21); however, Shrinivas *et al* (21) did not compare this combination with single drugs. The research conducted by Borle and Borle (22) hypothesized that the administration of different drugs through intralesional injections contributes to intensified fibrosis and significant trismus. The deteriorating effect of submucosal injections on this condition may be attributed to the repeated penetration of the needle into the soft tissues at various locations, clinical irritation caused by the injected drugs, and the progressive nature of the disease (22). To the best of our knowledge, there is a lack of published literature offering a universally accepted protocol and recommending an optimal pharmacological regimen for the treatment of OSMF. For patients with OSMF who are at the advanced stages of the disease and are undergoing complex surgical procedures, alternative options exist, such as the utilization of scalpel blades, electrocautery and laser therapy (23).

In the present study, the control group, who was administered oral medications, along with mouth opening exercises, also exhibited an improvement in signs and symptoms. Although the difference was not significant, it was less prominent when compared with the treatment with the intralesional injections of various drugs. The use of antioxidants containing lycopene can counteract the detrimental impact of free radicals via physical and chemical means, thereby safeguarding cellular constituents against impairment caused by highly reactive oxygen species. However, the efficacy of lycopene as a standalone therapeutic agent has been found to be suboptimal compared with combination therapies in terms of enhancing mouth opening (11). Previous research has shown a significant improvement in mouth opening with mouth exercises, which was not observed in the present study (24). This may be due to the non-performance of mouth-opening exercises regularly by patients despite repeated reminders.

The previous systematic review conducted by More *et al* (25) demonstrated that a comprehensive treatment approach involving the use of nutritional supplements and intralesional injection therapy was efficacious in the management of OSMF, as observed in the present study. However, it is imperative to investigate newer and more sophisticated approaches for the direct administration of therapeutic agents into affected areas to address the tendency towards fibrosis.

The present study had certain limitations, which should be mentioned. Sex differences could not be evaluated in the present study, as the disease was predominantly observed in the male population. As the investigation was performed on individuals from rural communities who had limited financial resources, mouth opening exercises were executed using traditional ice-cream sticks instead of mouth-exercising devices. Additionally, this particular study was conducted as a single-center study owing to ethical and financial constraints. It should be noted that the occurrence of fibrosis resulting from repeated intra-lesional injections, although not proven, was not assessed in the present study. Therefore, further long-term, multicenter, randomized controlled trials with long-term follow-up periods are warranted.

In conclusion, although there is no single treatment modality for the successful treatment of OSMF, the present study found that the intralesional injection of combination drugs, such as 2 ml placental extracts, 1 ml hyaluronidase, 1 ml dexamethasone, mixed with 1 ml of 2% lignocaine, along with SM fibro tablets twice daily, mouth opening exercises, massaging with 0.1% triamcinolone oromucosal paste twice daily and rinsing with benzydamine 0.15% mouthwash, is highly effective in improving mouth opening and the burning sensation in patients with grade 2 and 3 OSMF. A minimal relapse of 0.2 mm was observed after 1 year of follow-up. Future research is required however, to focus on conducting extensive clinical trials and longitudinal studies to validate the safety and efficacy of this treatment approach. In addition, it is crucial to focus on preventive measures by educating young individuals on the disease and on the risks of using tobacco products and betel quid.

## Figures and Tables

**Table I:** Demographic data and habits and complaints of the patients included in the study groups.

Demographics (n=137)					
Characteristic	Group 1 (n=32)	Group 2 (n=35)	Group 3 (n=35)	Group 4 (n=35)	P-value
No. of patients with grade 2 OSMF	21 (65%)	22 (62.8%)	21 (60%)	22 (62.8%)	0.971 (NS)
No. of patients with grade 3 OSMF	11 (35%)	13 (37.2%)	14 (40%)	13 (37.8%)	
Average age, years, mean ± SD	33.1±3.2	34.2±2.7	32.6±3.8	34.6±2.1	0.053 (NS)
Males	22 (69%)	20 (57%)	25 (71%)	26 (74%)	0.07 (NS)
Females	10 (31%)	15 (43%)	10 (29%)	9 (27%)	
Habits (n=137)					
Type of habit	Group 1 (n=32)	Group 2 (n=35)	Group 3 (n=35)	Group 4 (n=35)	Total ratio (%)
Tobacco (M/F)	20/00	16/00	23/00	23/00	82/00 (88/00%)
Betel quid (M/F)	02/10	04/15	02/10	03/09	11/44 (12/100%)
Patient complaints (n=137)					
Type of complaint	Group 1 (n=32)	Group 2 (n=35)	Group 3 (n=35)	Group 4 (n=35)	Total ratio (%)
White patch	15	20	19	21	75 (55%)
Reduced mouth opening	25	32	31	32	120 (88%)
Burning sensation	03	04	03	05	15 (11%)
Blood profiles (n=137)					
Blood parameter	Group 1 (n=32)	Group 2 (n=35)	Group 3 (n=35)	Group 4 (n=35)	Total ratio (%)
Increased ESR	0	1	1	1	3 (2%)
Low serum iron	1	2	2	0	5 (3.5%)
Low vitamin B12	0	1	2	1	4 (3%)

OSMF, oral submucous fibrosis; SD, standard deviation; NS, not significant; M, Male; F, Female; ESR, Erythrocyte sedimentation rate.

**Table II:** Comparison of mean mouth opening (in mm) at different time intervals in the study groups using ANOVA.

	Baseline (mean ± SD)	4th week (mean ± SD)	8th week (mean ± SD)	12th week (mean ± SD)	12th month (mean ± SD)	ANOVA between intervals (P-value)
Group 1	22.18±1.44	22.44±1.53	22.58±1.67	22.54±1.47	22.74±1.32	0.067
Group 2	22.08±1.51	24.80±1.68	27.01±1.83	28.03±1.83	26.41±1.78	0.001^b^
Group 3	22.15±1.35	24.04±1.39	25.84±1.58	27.26±1.59	26.76±1.54	0.001^b^
Group 4	22.32±1.36	25.40±1.61	27.79±1.73	29.62±1.88	29.33±1.65	0.001^b^
ANOVA between groups (P-value)	0.85	0.02^a^	0.003^a^	0.001^b^	0.001^b^	

^a^P<0.05, significant difference;

^b^P<0.001, highly significant difference. SD, standard deviation.

**Table III:** Intragroup analysis of mean mouth opening at the 12th week in the different study groups using Tukey's post-hoc analysis.

Intragroup comparison	P-value
Group 1 vs. group 2	0.0001^b^
Group 1 vs. group 3	0.0001^b^
Group 1 vs. group 4	0.0001^b^
Group 2 vs. group 3	0.131
Group 2 vs. group 4	0.01^a^
Group 3 vs. group 4	0.0001^b^

^a^P<0.05, significant difference;

^b^P<0.001, highly significant difference.

**Table IV:** Comparison of the mean scores of burning sensation at the different time intervals in study groups using the Kruskal-Wallis test.

Groups	Baseline (mean ± SD)	4th week (mean ± SD)	8th week (mean ± SD)	12th week (mean ± SD)	12th month (mean ± SD)	Kruskal-Wallis test between time intervals (P-value)
Group 1	3.09±0.68	3.09±0.68	2.62±0.66	2.15±0.44	1.34±0.65	0.001^b^
Group 2	3.11±0.63	3.11±0.63	2.28±0.45	1.25±0.44	0.82±0.61	0.001^b^
Group 3	3.20±0.63	3.20±0.63	2.31±0.47	1.45±0.50	0.45±0.50	0.001^b^
Group 4	3.37±0.59	2.37±0.59	1.42±0.50	0.42±0.50	0.22±0.42	0.001^b^
Kruskal-Wallis between groups (P-value)	0.272 (NS)	0.001^b^	0.001^b^	0.001^b^	0.001^b^	

^a^P<0.05, significant difference;

^b^P<0.001, highly significant difference. NS, not significant; SD, standard deviation.

**Table V:** Intragroup analysis of the mean scores of burning sensation at the 12th week in the different study groups using Dunn's post-hoc test.

Intragroup comparison	P-value
Group 1 vs. group 2	0.01^a^
Group 1 vs. group 3	0.001^b^
Group 1 vs. group 4	0.001^b^
Group 2 vs. group 3	0.12 (NS)
Group 2 vs. group 4	0.01^a^
Group 3 vs. group 4	0.01^a^

^a^P<0.05, significant difference;

^b^P<0.001, highly significant difference. NS, not significant.

## Data Availability

The datasets used and/or analyzed during the current study are available from the corresponding author upon reasonable request.
